# Head Position in Stroke Trial (HeadPoST) – sitting-up vs lying-flat positioning of patients with acute stroke: study protocol for a cluster randomised controlled trial

**DOI:** 10.1186/s13063-015-0767-1

**Published:** 2015-06-05

**Authors:** Paula Muñoz-Venturelli, Hisatomi Arima, Pablo Lavados, Alejandro Brunser, Bin Peng, Liying Cui, Lily Song, Laurent Billot, Elizabeth Boaden, Maree L. Hackett, Stephane Heritier, Stephen Jan, Sandy Middleton, Verónica V. Olavarría, Joyce Y. Lim, Richard I. Lindley, Emma Heeley, Thompson Robinson, Octavio Pontes-Neto, Lkhamtsoo Natsagdorj, Ruey-Tay Lin, Caroline Watkins, Craig S. Anderson, for the HeadPoST Collaborative Investigators

**Affiliations:** The George Institute for Global Health, University of Sydney and Royal Prince Alfred Hospital, Sydney, Australia; Vascular Neurology Program, Neurology Service, Department of Medicine, Clínica Alemana de Santiago, Universidad del Desarrollo, Santiago, Chile; Department of Neurological Sciences, Faculty of Medicine, University of Chile, Santiago, Chile; Department of Neurology, Peking Union Medical College Hospital, Beijing, China; Department of Neurology, Shanghai 85th Hospital of PLA, Shanghai, China; School of Health, University of Central Lancashire, Preston, UK; Nursing Research Institute, St Vincent’s Health Australia (Sydney) and Australian Catholic University, Sydney, Australia; Department of Cardiovascular Sciences and Leicester Cardiovascular Biomedical Research Unit, University of Leicester, Leicester, UK; Vascular and Neurology Emergency Service, Faculty of Medicine, Clinical Hospital of Ribeirão Preto, University of Sao Paulo, Ribeirão Preto, Brazil; Stroke Unit, State Third Hospital, Ulanbaatar, Mongolia; Stroke Centre, Kaohsiung Medical University Chung-Ho Memorial Hospital, Kaohsiung, Taiwan

**Keywords:** Cluster clinical trial, Head position, Ischemic stroke, Management, Nursing care, Outcomes, Stroke

## Abstract

**Background:**

Positioning a patient lying-flat in the acute phase of ischaemic stroke may improve recovery and reduce disability, but such a possibility has not been formally tested in a randomised trial. We therefore initiated the Head Position in Stroke Trial (HeadPoST) to determine the effects of lying-flat (0°) compared with sitting-up (≥30°) head positioning in the first 24 hours of hospital admission for patients with acute stroke.

**Methods/Design:**

We plan to conduct an international, cluster randomised, crossover, open, blinded outcome-assessed clinical trial involving 140 study hospitals (clusters) with established acute stroke care programs. Each hospital will be randomly assigned to sequential policies of lying-flat (0°) or sitting-up (≥30°) head position as a ‘business as usual’ stroke care policy during the first 24 hours of admittance. Each hospital is required to recruit 60 consecutive patients with acute ischaemic stroke (AIS), and all patients with acute intracerebral haemorrhage (ICH) (an estimated average of 10), in the first randomised head position policy before crossing over to the second head position policy with a similar recruitment target. After collection of in-hospital clinical and management data and 7-day outcomes, central trained blinded assessors will conduct a telephone disability assessment with the modified Rankin Scale at 90 days. The primary outcome for analysis is a shift (defined as improvement) in death or disability on this scale. For a cluster size of 60 patients with AIS per intervention and with various assumptions including an intracluster correlation coefficient of 0.03, a sample size of 16,800 patients at 140 centres will provide 90 % power (α 0.05) to detect at least a 16 % relative improvement (shift) in an ordinal logistic regression analysis of the primary outcome. The treatment effect will also be assessed in all patients with ICH who are recruited during each treatment study period.

**Discussion:**

HeadPoST is a large international clinical trial in which we will rigorously evaluate the effects of different head positioning in patients with acute stroke.

**Trial registration:**

ClinicalTrials.gov identifier: NCT02162017 (date of registration: 27 April 2014); ANZCTR identifier: ACTRN12614000483651 (date of registration: 9 May 2014). Protocol version and date: version 2.2, 19 June 2014.

**Electronic supplementary material:**

The online version of this article (doi:10.1186/s13063-015-0767-1) contains supplementary material, which is available to authorized users.

## Background

Stroke is a major global disease burden for which there are few proven treatment options. Acute ischaemic stroke (AIS) is the most frequent pathological subtype [1], where the likelihood of a patient’s having died or being dependent at 6 months is greater than 50 % [2]. In this disease, an occluded artery by in situ thrombus or embolism from a more proximal source (i.e., cardiac or extracranial vessels) impedes cerebral blood flow (CBF). The size and site of such an occlusion, as well as the efficiency of compensatory collateral blood flow, determine the extent of at risk (‘ischaemic penumbra’) and dead (‘infarcted’) brain [3]. As autoregulation is lost in the affected area, local CBF is considered to depend passively on mean systemic arterial blood pressure [4].

A simple way of potentially increasing CBF via the collateral circulation and into the ischaemic penumbra is to put the patient with AIS into a lying-flat (0°) head position. Several observational studies have used transcranial Doppler (TCD) to show that the lying-flat position is associated with an increase in CBF velocities within major cerebral arteries [5, 6]. Moreover, a significant increase in TCD-recorded mean flow velocity, and thus presumed CBF, has been recorded in the stroke-affected hemisphere, but not on the contralateral side, of patients with AIS who were positioned lying-flat (at 0° or 15°) compared with those positioned sitting-up (≥30°) [7]. However, the relevance of these changes to any improvement in clinical outcomes after AIS is uncertain at this time [8, 9].

In the subset of patients with mass effect caused by cerebral oedema in acute stroke, such as those with malignant middle cerebral artery infarction or primary intracerebral haemorrhage (ICH), sitting-up may improve the chances of a good outcome. Extrapolating from patients with acute brain injury, the authors of a systematic review of head positioning showed that intracranial pressure is decreased significantly when the head is elevated from 0° to 30°, whereas cerebral perfusion pressure is generally unchanged [10]. However, there appears to be little or no change in cerebral perfusion pressure reported in patients with different types of acute stroke [11, 12].

A common concern among clinicians is that positioning a patient lying-flat may increase the risk of aspiration pneumonia. The risk of pneumonia by aspiration of gastric contents is increased in the presence of dysphagia [13, 14] and where mechanical ventilation is required [15], but it is only in mechanically ventilated patients that the risk of pneumonia appears higher while they are lying-flat compared with sitting-up [16]. Although some clinical guidelines recommend that patients with stroke should be nursed with their head elevated to reduce the risk of aspiration pneumonia [17], the conclusion of a recent study was that avoidance of the lying-flat position over concerns of pneumonia may be unjustified, as the authors found a very low frequency (4.5-6 %) of pneumonia caused by lying-flat in patients with AIS after thrombolysis [18]. There is currently no clear evidence regarding the risks of aspiration pneumonia related to different head positions in nonventilated patients with acute stroke. Furthermore, side-lying and avoidance of feeding in these patients are likely to reduce such risks [19, 20].

Another argument against laying patients flat in bed is that it may delay mobilisation and rehabilitation. Even though a pilot phase study suggested that very early (<24 hours) rehabilitation was safe and associated with a nonsignificant improvement in function [21, 22], the main results of the pivotal A Very Early Rehabilitation Trial for Stroke (AVERT) showed that a high-dose very early mobilisation protocol that included frequent out-of-bed sitting, standing and walking activity was associated with a reduction in the odds of a favourable outcome at 3 months compared with usual care [23]. To date, there is no evidence to support intense very early mobilisation in the first 24 hours after the onset of acute stroke.

Regarding physiological parameters, although most patients do not experience clinically significant desaturation when their body position is changed, side-lying may reduce arterial oxygen saturation, particularly in patients with severe stroke associated with right hemiparesis and concomitant chest disease [24]. Moreover, stroke patients who are nursed in a sitting position may have higher arterial oxygen saturation levels than those in a supine position [24, 25]. Nevertheless, the results of the AVERT trial [23] suggest that increasing periods of sitting-up in the early phase of acute stroke may not necessarily improve outcome. The influence of changes in arterial oxygen saturation related to position on brain recovery and outcome after acute stroke remains to be confirmed.

We initiated the Head Position in Stroke Trial (HeadPoST) to determine the balance of risks and benefits associated with the lying-flat versus sitting-up head position in patients with acute stroke without a definite indication or contraindication to either intervention applied within the first 24 hours of admission to hospital. A cluster randomised design with the interventions under investigation applied as part of usual background nursing care was chosen to avoid contamination and maximise adherence, reliability and generalisability of the results. A crossover component is added to provide all hospital sites with a standardised change of policy, which will allow us to control for confounding factors that may be associated with differences in the organisation and levels of background care across hospitals.

### Aims

The primary aim of HeadPoST is to compare the effects of lying-flat (0°) with sitting-up (≥30°) in the first 24 hours of admission for patients presenting with AIS on death and functional recovery according to the modified Rankin Scale (mRS) score [26] at 90 days. This outcome pertains to the individual participant level.

The key secondary aims are to determine whether lying-flat (0°) is superior to sitting-up (≥30°) with regard to poor outcome (death and neurological impairment based on the National Institutes of Health Stroke Scale [NIHSS] [27]) at 7 days in patients with AIS, whether sitting-up (≥30°) is superior to lying-flat (0°) on early (death and neurological impairment at 7 days) and late outcomes (according to mRS scores at 90 days) and early neurological recovery (NIHSS at 7 days) in acute ICH, the effects of the two head positions on overall and cause-specific death separately by 7 and 90 days, serious adverse events (SAEs) and length of hospital stay.

## Methods/Design

### Trial design

**Fig. 1:**
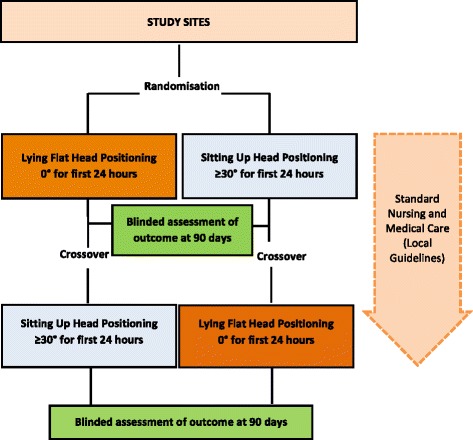
Trial schema

HeadPoST is an international, multicentre, prospective, cluster randomised, crossover, blinded outcome-assessed trial to be conducted through a global network of investigators, with hospitals as the unit of randomisation (clusters). All hospitals will participate in both the lying-flat (0°) and the sitting-up (≥30°) head position treatment phases. They will enrol and implement the intervention until the target number of patients is reached before crossing over to the opposite intervention. The study scheme outlines the flow and crossover of interventions (Fig. 1).

### Trial population

The trial will be conducted in approximately 140 hospitals (sites) in Australia, Brazil, Chile, China, Mongolia, Singapore, Taiwan and the United Kingdom in the first instance. Hospitals in other countries will be invited to join according to interest, feasibility and resources. Sites are required to fulfil certain eligibility criteria, including having an established acute stroke care program within a geographically defined area for the management of stroke patients (i.e., an acute stroke unit [ASU]) and a sufficient projected throughput of patients to ensure feasibility of recruitment within a short time frame.

### Consent process and participant inclusion and exclusion criteria

Each participating site must obtain written approval from its research ethics committee (REC) (e.g., institutional review board [IRB]), as well as from any other relevant regional or national bodies, before patient recruitment can commence. A list of all ethical bodies that have approved the study is provided in Additional file 1. A mixed consent process is proposed, according to local and/or national rules and regulations, as outlined in Table 1. Consent under the cluster guardian format or appropriate approval is necessary to prevent contamination of the intervention across patients nursed in closed proximity and by busy clinicians caring for multiple patients [28]. It is also likely to avoid responder bias in patients (or their surrogates) as a result of potentially thinking that they have received nonstandard care [29]. Under the guardian consent process, all eligible patients will receive the intervention as standard of care and will be provided with an approved Patient Information Sheet (PIS) and Consent Form as soon as practical after admission. According to the local REC-approved consent process, patients’ written consent to be either included or excluded—opt-in or opt-out patient consent, respectively—will be obtained before enrolment to collect their medical and personal information and to contact them again for follow-up at 90 days. These patients will also be able to formally opt out at any stage of the study.

**Table 1:** Consent options for the HeadPoST trial

*Hierarchy of consent options proposed in this study*	
1.	Cluster guardian consent or appropriate approval (e.g., signed by general manager or chief executive of hospital or by head of neurology/stroke department) for the randomised head position to be the usual nursing care for patients with acute stroke, obtained before commencement of the study
With any of the following:	
2.	*a. Recommended*: Opt-out consent obtained from patients for the collection of data through in-person assessment and extraction of information from medical records during the hospital stay and follow-up, and release of personal information to allow centralised follow-up at 90 days after initial admission to the hospital for research purposes
	*b. Alternative*: Individual patient consent for collection of in-hospital data and for release of personalised information for research purposes to allow centralised follow-up at 90 days after the initial admission to the hospital

The patient eligibility criteria have been kept simple to facilitate the implementation of the randomised head position as a standard of care and for evaluation of the treatment effect in a broad range of patients. Consecutive eligible patients with acute stroke will be approached to participate in the trial. The patient inclusion and exclusion criteria are given below.

#### Inclusion criteria

All patients are eligible for the allocated intervention if, at the time of presentation to the hospital, they meet each of the following criteria:

1. Adult aged 18 years or older (a younger age of 16 years may be used in some countries)
2. Presumed clinical diagnosis of acute stroke (i.e., with a persistent neurological deficit on presentation)
3. Either present directly, are transferred from another hospital or have had an in-hospital event

#### Exclusion criteria

Patients are to be excluded from the allocated intervention if at the time of presentation they meet any of the following criteria:

1. A resolved transient ischemic attack (i.e., brief neurological symptoms that are judged to have completely resolved upon presentation)
2. A definite clinical contraindication or indication for either the sitting-up or lying-flat head position
3. A significant medical condition that takes priority in care and where adherence to the randomised head position is not possible on another ward or department of the hospital, such as for haemodialysis or surgery (e.g., carotid endarterectomy, haematoma evacuation)
4. Not consenting to participate in HeadPoST
5. Previously enrolled in HeadPoST

### Randomisation

The unit of randomisation is the hospital. A statistician not otherwise involved in the trial will generate the randomised allocation sequence and treatment group assignment of either lying-flat (0°) or sitting-up (≥30°) as the first intervention before crossover to the other intervention. Participating sites will be stratified according to country, and the allocation sequence will be concealed until the interventions are assigned.

### Interventions

The allocated interventional head position is to be applied to all consecutive presenting (or in-hospital) eligible patients as soon as possible after the clinical diagnosis of stroke is made. This pertains to both the cluster and patient levels.

#### Lying-flat (0°)

Patients will be positioned lying-flat (0°) as soon as possible after presentation to the emergency department (ED) or other assessment area, unless there is a specific contraindication. Patients are to remain in this position for at least 24 hours. Patients can have a swallow screen and/or swallow assessment and can be allocated nil-by-mouth (NBM), nasogastric feeding, modified diet or normal diet, according to local protocols. However, it is recommended that nasogastric feeding be undertaken only as bolus feeds (i.e., not continuous) and with the patient in a sitting position for short periods as necessary to reduce the risk of aspiration [19]. All other feeding can be undertaken with the patient lying down, unless this position is definitely not tolerated, in which case the patient can sit up for no more than 30 minutes. It is possible for patients to eat on their side in the flat position, as swallowing is an active process that is not dependent on gravity. Patients should have no more than three breaks of 30 minutes from a flat position in the first 24 hours, and breaks should not to be grouped together (i.e., no back-to back breaks are permitted). All patients should be toileted in bed or in a commode near the bed, where possible. Gentle graded mobilisation with toilet privileges, and elevation of the head, can occur after the first 24 hours. The head may be raised gradually after 24 hours of lying-flat, if necessary, but patients with moderate to severe neurological deficits may have the flat position maintained for longer. Patients are to be mobilised according to local stroke care guidelines.

#### Sitting-up (≥30°)

Patients will be positioned sitting-up with head elevated at least 30° by raising the head of the bed or using extra pillows, whichever is more appropriate, immediately upon presentation to the ED, and they are to remain in this position for at least 24 hours. Patients can have a swallow screen and/or swallow assessment and can be allocated NBM, nasogastric feeding, modified diet or normal diet, according to local protocols. In the unlikely situation that a patient has to be nursed with the head lowered (e.g., to perform computed tomography), the same time-off restrictions are applied (i.e., no more than three breaks of 30 minutes in a lying-flat (0° or <30°) position in the first 24 hours and no break periods to be grouped together). Feeding may commence after patients have passed an appropriate swallowing screening test or swallowing assessment. Those patients will be allowed mobilisation according to local guidelines.

#### Background care

All patients with acute stroke should be managed by a dedicated team in an ASU (or high-dependency unit or intensive care unit) during the period of the intervention. Their management should be best practice standard of care according to regional guidelines, including use of a swallowing screen or swallowing assessment before any feeding is initiated.

### Trial outcomes

Trial outcomes pertain to patient-level data. The overall primary outcome of HeadPoST is a shift (improvement) in death and disability according to an independent telephone assessment using the mRS [30] at 90 days. The secondary outcomes are death or dependency measured by a shift in NIHSS [27] at 7 days, death within 90 days, length of hospital stay, health-related quality of life according to the 5-dimension European Quality of Life Scale (known more commonly as the EQ-5D) and pneumonia according to standard criteria involving a set combination of symptomatology and radiological signs [31].

### Data collection and follow-up

Sites should record details of all patients admitted with acute stroke, and all patients should be placed in the randomised head position unless there is a clear contraindication (e.g., cardiac or respiratory failure). All patients will be approached for enrolment for data collection within the trial by site study coordinators and investigators. Brief demographic details of all stroke patients who are approached but who are not enrolled, and the reason for nonenrolment, will be recorded on a screening and enrolment log to determine selection bias. For safety analysis, sites are requested to provide information on any death known among registered nonenrolled patients during follow-up. To assist the implementation of the intervention at each site, information will be gathered on the organisational structure of the site and the various clinical wards that will be involved in the intervention. Each site’s lead investigator is required to complete a hospital organisation questionnaire, developed based upon previous surveys in this field [32–34], to assist the regional coordinating centre (RCC) in preparing the training and site initiation visit (see Additional file 2).

The goal of the main assessments of participants in the first 24 hours will be to promote adherence to the allocated head position. A monitoring chart will be maintained by clinical staff to record the duration in the allocated head position, but importantly also the time spent out of position with a description of the reason. Basic physiological parameters will also be recorded. Sites are also required to collect a limited amount of data on patients at the time of admission (day 1) and separation (day 7 or at discharge, transfer or death, if earlier), as well as all SAEs, including death, until 90 days.

Appropriately trained outcome assessors, who are kept blind to the management of patients, will use a script to conduct a telephone assessment of health and functioning at 90 days.

### Serious adverse events

The SAEs are defined as recommended by the World Health Organisation International Drug Monitoring Centre. The mechanisms for reporting and notifying SAEs are based on the guidelines adopted by the International Conference on Harmonisation of Technical Requirements for Registration of Pharmaceuticals for Human Use – Good Clinical Practice (ICH-GCP) [35] and refer to those related to each patient recruited into the study from the period of enrolment until the assessment at 90 days. The international coordinating centre (InCC) will closely monitor all SAEs for any relationship to the study procedures and protocol and for any clustering of events at a particular site. The protocol will be amended or the trial stopped early if an excess of a particular SAE appears to be protocol-related, including pneumonia, neurological deterioration and heart failure. In addition, the InCC will submit all SAEs to the appointed independent Data and Safety Monitoring Board (DSMB) for regular review and, if needed, outside the planned safety and interim analysis meetings.

### Quality control measures

RCCs will be set up in the various countries to facilitate the compliance and translation of the protocol to meet local regulations. RCC staff will receive training and assistance from the InCC in the setup and documentation required for the study in accordance with the Declaration of Helsinki and ICH-GCP standards. There will be regular meetings and/or teleconferences between the RCC and InCC staff. The InCC will provide standard operating procedures (SOP) to the RCCs and sites to assist with compliance with the protocol. Manuals and guidelines will be developed by the InCC in liaison with the operations committee. Training will be provided in both online and face-to-face meetings undertaken by staff from the RCCs using the training materials developed specifically for the study.

#### Monitoring of sites

RCC-based clinical research monitors will perform online and on-site data verification and monitor the conduct of the study. A nominated ‘local champion’ at each site will assist in assessing compliance with the head position by providing ongoing training, solutions to local barriers and ad hoc checking of the positioning procedure and recordings in the ED, the ASU ward, and any relevant wards that take stroke patients in the first 24 hours. In addition, experienced RCC research staff will undertake quality control activities necessary for conduct of the trial. Monitoring serves to confirm adherence to the protocol and guidelines, relevant local and regional ethical requirements, and data accuracy and quality.

### Coenrolment

As the HeadPoST trial is a cluster randomised controlled trial of an organizational change, there are no methodological contraindications to coenrolment of patients into individual patient randomised controlled trials. Although the aim is to recruit all consecutive stroke patients, allowance is made for coenrolment of patients into individual patients’ randomised pharmaceutical investigational or rehabilitation trials, or into observational registry studies, provided that this is acceptable to participants (who will likely have to complete additional follow-up requirements), the local REC, cluster guardians and competing trial sponsors/chief investigators. As most conventional individual patient randomised clinical trials recruit only a minority (e.g., 2-10 %) of all patients, it is anticipated that only a few patients could be enrolled in multiple research studies or clinical trials. If coenrolment is unacceptable and patients are included in another trial, then an explanation is to be given in the screening log regarding why selected patients were excluded from HeadPoST.

### Statistical considerations

#### Sample size

In patients with AIS, lowering the head from 30° to 15° or 0° has been associated with up to 12 cm/s increases in mean CBF in the middle cerebral artery on TCD [12, 36–38]. Other studies have shown that a 1 cm/s increase in CBF is associated with a 0.7-point reduction in NIHSS score [9] and a 16 % reduction in death or dependency based on the mRS [39], whereas the score distributionin the sitting-up head position has been reported to be 0 (18 %), 1 (18 %), 2 (16 %), 3 (15 %), 4 (12 %), 5 (12 %) and 6 (death, 9 %) [40]. For a cluster size of 60 patients with AIS for each intervention (i.e., lying-flat or sitting-up), and assuming 5 % crossover and 10 % dropout rates in each hospital, recruitment failure in 10-15 % of hospitals, and an intracluster correlation coefficient of 0.03 (conservatively estimated from an intracluster correlation coefficient of 0.018 in another cluster controlled trial [41] undertaken across 19 ASUs in New South Wales, Australia), a sample size of 16,800 patients with AIS at 140 sites will provide 90 % power (α 0.05) to detect at least 16 % improvement (shift) in death and disability on the mRS at day 90 in the ordinal logistic regression analysis [42, 43]. There will also be 90 % power to detect at least 16 % improvement (shift) in neurological function on the NIHSS at day 7, at least 30 % reduction in death and at least a 2-day reduction in length of stay for such patients.

For patients with acute ICH, the cluster size will be smaller and may vary across sites (10-30 %), particularly between China and elsewhere [1, 44], depending on the frequency of ICH. Assuming a recruitment of 10 ICH patients on average per site for each intervention period, a sample size of 2,800 patients with ICH at 140 sites will provide 90 % power (α 0.05) to detect at least 25 % improvement (shift) in death or disability associated with the sitting-up head position. Moreover, there will be 90 % power to detect at least 25 % improvement in NIHSS at day 7, at least 33 % decrease in death and at least a 2-day reduction in length of stay for these patients.

The power of the trial is derived from having a very large number of clusters, which we consider achievable because the workload at each site will be kept low and for a short period of time. The inflation of the cluster size and the number of clusters are being done to take account of stroke mimics, poor recruitment and quality issues. An overall target of 70 patients in each intervention group is derived from the requirement of 60 and 10 with AIS and ICH, respectively. For the smaller ASUs with fewer than 200 people admitted with stroke per annum, the sample size will likely be achieved over 4 to 5 months, so, taking account of the crossover and 90-day follow-up, the total duration of the study is approximately 12 months. For large ASUs, and especially for the hospitals in China with over 1,200 stroke admissions per annum, the required total number of 140 (2×70) patients to be recruited is likely achieved just over several weeks, for a study duration of 4 to 5 months.

#### Data analysis

We will analyse patients in the treatment group to which they are allocated according to the intention-to-treat principle. We will compare patients allocated to lying-flat with those allocated to sitting-up. The primary analysis will be unadjusted, but adjusted analyses can also be carried out on the primary and secondary outcomes if required. All analyses will be adjusted for clustering within sites. No adjustment for multiplicity is planned, as there are only a small number of prespecified efficacy outcomes being investigated. All analyses will be undertaken at the patient level on an intention-to-treat basis, as defined by allocated head position at each hospital, using generalised estimating equations (GEE) or random-effects regression to account for clustering.

The primary outcome of death or disability according to the mRS [26] score at 90 days will be analysed by means of GEE for ordinal data (i.e., the natural extension of ordinal logistic regression [‘shift’] analysis for clustered data), in which mRS as a dependent variable with seven levels (ranging from 0 [no residual symptoms] to 6 [death]). The secondary outcome of the NIHSS [27] score at 7 days will also be analysed similarly, as the NIHSS [27] is considered as an ordinal endpoint with seven levels [45]. Binary secondary outcomes will be analysed by means of standard GEEs with a logistic link and/or time-to-event type endpoints using the Cox model with a sandwich formula [46]. For continuous outcomes, a random intercept linear regression model will be used. Descriptive statistics will be provided for safety data. The number of patients reporting any SAEs and the occurrence of specific SAEs will be tabulated. Tests of a treatment effect on specific SAEs may be attempted by means of a χ^2^ test adjusted for clustering. Subgroup analyses will also be conducted to investigate the effects of the interventions in different pathological subtypes of AIS, defined as small vessel ‘lacunar’, large vessel, cardioembolic and other.

### Process evaluation

By exploring the way in which each of the study interventions is implemented, it will be possible to provide insights into why each was successful and how each one can be optimized, and the reasons for the failure of an intervention or for unexpected consequences can be analysed [47–49]. Furthermore, assessment of implementation is essential for analysing the internal and external validity of interventions [49].

Among a set of key implementation measurements identified [50], those most relevant to HeadPoST include assessment of the following:

1. *Fidelity*: By monitoring the positioning policy that sites are implementing
2. *Dosage*: By assessing for how many periods and for how long patients have been in a particular head position during the first 24 hours
3. *Quality of intervention delivered*: By monitoring the delay until the start of the intervention after admission
4. *Program reach*: Percentage of eligible population who participated, derived from data on enrolment and screening logs
5. *Adaptations to program during implementation*: By recording feedback from investigators

In addition, semistructured interviews with selected health care providers will be conducted by local research staff during the site initiation process to assess any potential barriers and their solutions for delivering the implementation, as barrier assessment is proven to promote clinician behaviour change [51]. Such data will potentially optimise the implementation process during the trial, as well as inform strategies for the potential rollout of the intervention after the trial.

### Data management

The internet-based data management system will be managed at the InCC, which has extensive experience in clinical trial data capture and security. The InCC has in place system security SOP with VeriSign SSL digital certification and an encrypted HTTPS connection (IT-SOP-105 v1.4). Registration and data entry will be performed at the participating sites via the password-protected, encrypted HTTPS connection. Only staff listed in the delegation log will be given unique individual passwords to access the internet-based data management system. This system has been developed at the InCC for data capture. The data variables will have logic checks within the acceptable ranges and mandatory fields to ensure accuracy and reduce missing data. Reports and data query management will also be included in the system to assist with centralised online monitoring by the InCC and the RCC.

Paper case report forms will be provided for sites preferring to use these for the initial collection of data. These forms will be used as source documents and will need to be signed and dated by the investigator completing the form. All computerised forms will be electronically signed (by use of the unique password) by the authorized study staff, and all changes made following the initial entry will have an electronic dated audit trail. It is required that the collection of data and transfer of information for the 90-day follow-up assessment must be approved by the local IRB at each site.

### Confidentiality and privacy

Every precaution will be taken to respect the privacy of patients in the conduct of the study. To maintain patient confidentiality, only deidentified data will be used for statistical analyses and publication of results. However, as part of the centralised follow-up service, the InCC at The George Institute for Global Health (Sydney, Australia) and the RCCs will use contact sources recorded by the sites to undertake the 90-day assessment. Only names, telephone numbers, next of kin and contact details of a patient’s general practitioner will be sent to the RCC to undertake the follow-up assessment. The information will be encrypted and password-protected before being sent by email in batches. This information will be included in the PIS. In the course of monitoring data quality and adherence to the study protocol, the monitor will refer to medical records at the participating hospital. This information will be included in the PIS. All individual and site information will be deidentified in reporting data and results to protect the confidentiality of participants.

## Discussion

If positioning the patient in the acute phase of stroke has a significant beneficial effect on outcome, there is potential to have a major public health impact for a widely generalisable, affordable health care intervention. In the setting of AIS, the principal therapeutic approach is to restore antegrade perfusion within the ischaemic territory through early use of recombinant tissue plasminogen activator or a mechanical endovascular treatment, but both approaches are limited by access barriers and potential harms. Another potential therapeutic approach is to augment cerebral perfusion through three anatomical systems in the brain: large artery communications between the intra- and extracranial circulation, the circle of Willis, and leptomeningeal anastomotic channels [52]. The extent of leptomeningeal collateral vessels, as visualised by computed tomography with angiography, has been shown to be associated with outcome in AIS [53], and the presence of good collateral circulation determined by conventional cerebral angiography indicates a good prognosis after endovascular treatment for AIS [54]. Although increasing industry attention is being focused on the use of partial aortic occlusion pumps or external counterpulsation to increase CBF [55, 56], a far simpler approach to enhancing the cerebral collateral circulation is through lying patients flat in the hyperacute phase of AIS.

Although sitting patients up is standard policy for patients with acute stroke in most Western countries, an increasing number of ‘early adopter’ stroke centres (e.g., Switzerland) have introduced the lying-flat position for patients with AIS, in either all or specific subtypes of patients (i.e., occlusion of large proximal cerebral vessels) on the basis of encouraging data derived from small observational studies demonstrating increased CBF on TCD. Conversely, in low-income settings and/or countries, where most of the global stroke burden exists, the lying-flat position is widely applied because of the use of simple, nonmechanical beds. Taken together with other geographic variations in nursing practices and hospital care policies, the manner in which patients with acute stroke are nursed could be critical to rates of pneumonia.

In the absence of randomised trial evidence, there will be ongoing variation in opinion and policy over the most appropriate patient position in the acute phase of both the AIS and ICH forms of acute stroke. Such a low-cost and widely applicable policy regarding the position of the patient—lying-flat versus sitting-up—has potential for having a significant health impact in this major disease.

HeadPoST has been designed to determine the efficacy and safety of a simple nursing intervention in patients with acute stroke. The trial uses broad inclusion criteria and will be conducted across different health care settings to support the generalisability of results. The study aims to provide reliable evidence on the optimal head position to inform policy in the management of patients in the initial 24 hours following acute stroke.

### Administrative information

#### Steering committee

The study will be overseen by an international steering committee (SC) comprised of experts in the fields of stroke, neurocritical care, neurology, geriatrics, cardiovascular epidemiology and clinical trials. The SC will consist of regional country leaders and grantholders.

The following are the SC members: Professor Gillian Mead (Chair), University of Edinburgh, UK; Professor Craig Anderson (Deputy Chair and Principal Investigator), University of Sydney, Australia; Associate Professor Maree Hackett (Chief Investigator), University of Sydney, Australia, and University of Central Lancashire, Preston, UK; Associate Professor Laurent Billot, University of Sydney, Australia; Professor Hisatomi Arima (Chief Investigator), University of Sydney, Australia, and Shiga University of Medical Sciences, Otsu, Japan; Professor Pablo Lavados and Dr Verónica Olavarría, Clínica Alemana de Santiago, Universidad del Desarrollo, Santiago, Chile; Professor Sandy Middleton, Australian Catholic University and St Vincent’s Health Australia, Sydney, Australia; Professor Caroline Watkins, University of Central Lancashire, Preston, UK; Professor Thompson Robinson, University of Leicester, UK; Professor Liying Cui and Professor Bin Peng, Peking Union Medical College Hospital, Beijing, China; Professor Octavio Pontes-Neto, University of Sao Paulo, Brazil; Dr Lkhamtsoo Natsagdorj, Stroke Unit, State Third Hospital, Ulanbaatar, Mongolia; Professor Ruey-Tay Lin, Stroke Centre, Kaohsiung Medical University Chung-Ho Memorial Hospital, Kaohsiung Taiwan; and Professor Tsong-Hai Lee, Linkou Chang Gung Memorial Hospital, Taoyuan, Taiwan.

#### Responsibilities of the steering committee

The SC will have overall responsibility for the execution of the study protocol, data collection and analysis plan, and publications. The SC has the right to appoint new members and coopt others to add to the integrity of the conduct of the study and analyses.

#### Advisory committee

The following are the members of the advisory committee: Associate Professor Stephane Heritier, Monash University, Melbourne, Australia; Dr Emma Heeley, University of Sydney, Australia; Professor Richard Lindley, University of Sydney, Australia; Associate Professor Stephan Jan, University of Sydney, Australia; Professor Mark Woodward, University of Sydney, Australia; Elizabeth Boaden, University of Central Lancashire, Preston, UK; Dr Alejandro Brunser, Clínica Alemana de Santiago, Universidad del Desarrollo, Santiago, Chile.

#### Coordinating centres

Central international coordination will be handled at The George Institute for Global Health, Sydney, Australia, and together with RCCs established and located in Beijing, China; Preston, UK; and Santiago Chile.

#### International coordinating centre

The InCC is supported by key grantholders and project staff. It is responsible for day-to day management of the study, data and project management, committee coordination, assistance with ethics committee applications, protocol and procedures for training of participating sites, overseeing of initiation visits and activation of participating sites, monitoring of data quality and adherence to protocol, adherence to applicable guidelines and regulations, and preparation of study data for analysis and publication.

#### Regional coordinating centres

The RCCs are established in China, the United Kingdom and South America with responsibilities for providing advice to the InCC on regional issues relevant to the setup, translation and management of the study. In addition, working with the InCC, they will provide assistance and support in obtaining REC approvals; training and activating sites; and monitoring study progress at participating sites in their region, including data quality and adherence to the study protocol. The RCCs also will assist in identifying and overseeing the centralised follow-up assessment process for their regions.

### Data and Safety Monitoring Board

The DSMB will review the safety, ethics and outcomes of the study. It is independent from the sponsor and has no competing interests. DSMB members will monitor blinded responses of variables and SAEs for early dramatic benefits or potential harmful effects.

A charter that will outline member responsibilities, procedures and confidentiality will govern the DSMB. The following are the members of the DSMB: Professor Robert Herbert (Chair), Neuroscience Research Australia, University of New South Wales, Sydney, Australia; Associate Professor Christopher Chen, National University of Singapore, Singapore; and Professor Anne Forster, Bradford Institute for Health Research, Bradford, UK.

A DSMB will also review unblinded data at regular intervals during follow-up and will monitor neurological and functional changes (between the two groups), as well as dropout and event rates. These DSMB members will use the approach developed by Sir Richard Peto for safety monitoring and will provide reports to the InCC on recommendations to continue or temporarily halt recruitment into the study.

### Writing committee

Publication of the main reports derived from the study will be in the name of the HeadPoST Collaborative Investigators. Full editorial control will reside with a writing committee approved by the SC. Investigators have the right to publish or present the results of the study. However, as this is a multicentre study, investigators must agree not to publish or publicly present any interim results of the study without the prior written permission of the SC. Investigators must further agree to provide the SC at least 30 days’ prior notice of any submission for publication or presentation for review, copies of abstracts or manuscripts (including, without limitation, text and PowerPoint presentation slides and any other texts of translations or medial presentations) that report any study results. Authors of publications must meet the International Committee of Medical Journal Editors criteria for authorship.

## Trial status

Ethics committees have granted permission for the study to commence across hospitals in Australia, Brazil, Chile, China and the United Kingdom. Patient enrolment commenced in February 2015. As of 2 June 2015, 435 patients have been enrolled at 14 sites.

## Additional files

Additional file 1:
**List of full names of the ethics committees in the various countries and study sites that have approved the trial as of 11 May 2015.**
Additional file 2:
**Hospital Organisation Questionnaire.** Description: Developed questionnaire to capture baseline stroke care organisational structure of the various clinical wards within the study sites.

## Abbreviations

AIS: Acute ischaemic stroke
ASU: Acute stroke unit
AVERT: A Very Early Rehabilitation Trial for Stroke
CBF: Cerebral blood flow
DSMB: Data and Safety Monitoring Board
ED: Emergency department
GEE: Generalised estimating equation
HeadPoST: Head Position in Stroke Trial
ICH: Intracerebral haemorrhage
ICH-GCP: International Conference on Harmonisation of Technical Requirements for Registration of Pharmaceuticals for Human Use – Good Clinical Practice
InCC: International coordinating centre
IRB: Institutional review board
mRS: Modified Rankin Scale
NBM: Nil-by-mouth
NIHSS: National Institutes of Health Stroke Scale
PIS: Patient Information Sheet
RCC: Regional coordinating centre
REC: Research ethics committee
SAE: Serious adverse event
SC: Steering committee
SOP: Standard operating procedures
TCD: Transcranial Doppler
