# Supplementary material for: Head Position in Stroke Trial (HeadPoST) – sitting-up vs lying-flat positioning of patients with acute stroke: study protocol for a cluster randomised controlled trial
Source: Trials. 2015 Jun 5;16:256. doi: 10.1186/s13063-015-0767-1 (PMC4460701; doi:10.1186/s13063-015-0767-1)
Supplement: Additional file 1: — List of full names of the ethics committees in the various countries and study sites that have approved the trial as of 11 May 2015. [file 13063_2015_767_MOESM1_ESM.pdf]

Ethics Approvals obtained as of 11 May 2015

Country	Name of Committee
**Australia**	
Central Approval	Human Research Ethics Committee Sydney Local Health District Ethics Review Committee RPAH Zone.
Local Site Approval	Royal Prince Alfred Hospital, Research Governance
	Concord Repatriation General Hospital, Research Governance
	Human Research Ethics Committee Calvary Health Care Bruce
**Brazil**	
Central Approval	Not required
Local Site Approvals	CEP Hospital das Clínicas da Faculdade de Medicina de Ribeirão Preto da USP
	CEP Hospital Municipal São José, HMSJ Joinville, SC
**Chile**	Comité de Ética Científico Servicio de Salud Metropolitano Sur Oriente
	Comité de Ética Científico Servicio de Salud Metropolitano Oriente
	Comité de Ética Científico Servicio de Salud Araucanía Sur
	Comité de Ética de la investigación del Servicio de Salud Valdivia
	Comité de Ética Científico Servicio de Salud Iquique
	Comité de Ética de la Investigación Facultad de Medicina Clínica Alemana de Santiago Universidad del Desarrollo
	Comité de Ética Científico Clínica Dávila
	Comité de Ética Científico Servicio de Salud del Bio Bio
**China**	
Central Approval	The EC of The Hospital of Peking Union Medical College
Local Site Approval	The EC of Yutian County Hospital
	The EC of Beijing Pinggu Hospital
	The EC of Beijing University Shougang Hospital
	The EC of Bethune international Heping Hospital
	The EC of Cangzhou Cenral Hospital
	The EC of Central Hospital of Shanghai Changning District (Tongren Hospital)
	The EC of Chifeng College affiliated hospital
	The EC of Dalian Third People's Hospital
	The EC of Dongguan People's Hospital
	The EC of Dunhua City Hospital
	The EC of Huizhou City 3rd People's Hospital
	The EC of Liaoning Province People's Hospital
	The EC of Nanpi County Hospital
	The EC of People's Hospital of Hejian City
	The EC of Qilu Hospital of Shandong University
	The EC of Qinhuangdao Harbour Hospital
	The EC of Shanghai 85 Hospital
	The EC of Shijiazhuang City Central Hospital
	The EC of The 2nd Affiliated Hospital of Suzhou University
	The EC of The 2nd Hospital of Shandong University
	The EC of The 2nd Hospital of Hebei Medical University
	The EC of The 3rd Hospital of Hebei Medical University
	The EC of The Affiliated Hospital of Shandong University (Qingdao)
	The EC of The Chinese PLA No.263 Hospital
	The EC of The First Affiliated Hospital of Wenzhou Medical College

	The EC of The General Hospital of Yangquan Mining Limited liability company
	The EC of The First Hospital of Harbin Medical University
	The EC of The second Hospital of Nanchang
	The EC of The third Hospital Affiliated to Guangzhou Medical University
	The EC of Wafangdian 3rd hospital
	The EC of Xuanwu Hospital Affiliated to Capital Medical University
	The EC of Yongjia County People's Hospital
	The EC of Zhucheng TCM Hospital

Taiwan

Central Approval	Not required
Local Site Approval	Chang Gung Medical Foundation Institutional Review Board

United Kingdom

Central Approval	Wales Research Ethics Committee 5, Bangor
Local Site Approval	Not required
