# Supplementary material for: Head Position in Stroke Trial (HeadPoST) – sitting-up vs lying-flat positioning of patients with acute stroke: study protocol for a cluster randomised controlled trial
Source: Trials. 2015 Jun 5;16:256. doi: 10.1186/s13063-015-0767-1 (PMC4460701; doi:10.1186/s13063-015-0767-1)
Supplement: Additional file 2: — Hospital Organisation Questionnaire. Description: Developed questionnaire to capture baseline stroke care organisational structure of the various clinical wards within the study sites. [file 13063_2015_767_MOESM2_ESM.pdf]

HeadPoST
Head Position in Stroke Trial

Hospital organisation questionnaire

Hospital details

1. Is the hospital a teaching hospital? ☐ Yes ☐ No

2. Number of beds in the hospital? |_|_|_|_|_| beds

3. Location of the hospital: *(tick one answer)*
   - ☐ Metropolitan / urban
   - ☐ Semi-metropolitan / semi-urban
   - ☐ Rural / countryside

4. Does the hospital have an intensive care unit? ☐ Yes ☐ No

5. Does the hospital maintain a stroke database? ☐ Yes ☐ No

6. Does the hospital have onsite specialist physicians responsible for stroke patients? ☐ Yes ☐ No

7. Does the hospital use telemedicine links to smaller hospitals? ☐ Yes ☐ No

8. Does the hospital have transfer protocols for acute stroke treatment or care with other hospitals? ☐ Yes ☐ No

9. Does the hospital have a dedicated, multidisciplinary stroke team? ☐ Yes ☐ No

Stroke pathways

10. Does the city have an emergency call service? *(e.g. 9-1-1, or 0-0-0, or 9-9-9)* ☐ Yes ☐ No

11. Are ambulance services organised for direct transfer to the hospital of suspected stroke patients? *(tick one answer)*
    - ☐ Yes
    - ☐ No
    - ☐ No but, there is agreement to bypass the hospital for another stroke specific service
    - ☐ Unsure

12. What proportion of stroke patients arrive at the hospital by ambulance? |_|_| %

13. What proportion of stroke patients arrive within 4 hours of the onset of symptoms? |_|_| %

14. Is the ED contacted / notified of an acute stroke patient by the ambulance service before arrival at the hospital? ☐ Yes ☐ No

15. Do stroke patients have a fast track access to brain imaging from the ambulance to the radiology department? ☐ Yes ☐ No

16. Is there an acute "code stroke" pager system for potential thrombolysis patients? ☐ Yes ☐ No

17. Does the hospital manage complex strokes that require neurosurgical intervention? ☐ Yes ☐ No

18. Which team usually manages stroke patients at the hospital? *(tick one)*
    - ☐ General medical team
    - ☐ Stroke geriatric team
    - ☐ General geriatric team
    - ☐ Stroke neurology team
    - ☐ General neurology team
    - ☐ General practitioner/visiting medical officers

19. What proportions (%) of acute stroke patients are admitted to the following units or departments in the hospital? *(answer 0 to all that is not applicable)*

Short stay ward in emergency department	|_|_|_|	%
Intensive care unit	|_|_|_|	%
Specialised stroke care unit	|_|_|_|	%
Neurology ward	|_|_|_|	%
Neurosurgical ward	|_|_|_|	%
Geriatric ward	|_|_|_|	%
Geriatric rehabilitation ward	|_|_|_|	%
General medical ward	|_|_|_|	%
Other	|_|_|_|	%

Emergency Department (ED)

20. Number of beds in ED? |_|_|_|_|_| beds

21. Average number of patients seen in ED per month? |_|_|_|_|_|_| patients

Generally, what is the number of patients to one staff? *(answer 0 if not applicable)*

22. Number of patients to one nurse |_|_|

23. Number of patients to one health assistant |_|_|

24. Select what best describes the ED at the hospital:

☐ Dedicated ED

☐ No dedicated ED but a designated "emergency area" in a small hospital

a. If dedicated ED, does it have: *(tick all that apply)*

☐ an intensive care unit?
☐ a high dependency or acute medical unit (AMU)?
☐ a separate resuscitation area?
☐ a short stay ward?

25. Are there current ED clinical pathway/checklist/protocols for ischaemic stroke patients for:

☐ Rapid triage and treatment for acute stroke?
☐ Early transfer to another hospital?

26. Do you have a clinical pathway/checklist for patients with acute intracerebral haemorrhage? ☐ Yes ☐ No

27. What proportion of stroke patients stay in the ED beyond 4 hours? |_|_| %

28. Are there medical staff from the stroke service (specialist, fellow or registrar) available in ED? ☐ Yes ☐ No

a. If YES:

☐ Availability on site 24/7
☐ Monday to Friday during working hours (8-5), on-call after hours
☐ Designated phone or telemedicine support only
☐ Other, please specify: __________________

b. If NO stroke service available in ED, acute management advice primarily provided by:

☐ General Physician
☐ Emergency Medicine Specialist
☐ General Practitioner or Career Medical Officer
☐ Other, please specify: ___________________

29. What area in the ED are stroke patients mostly assessed? *(tick one)*

☐ General area
☐ Dedicated area for stroke patients
☐ Dedicated resuscitation area
☐ Short stay ward
☐ Other, please specify: ___________________

Imaging and investigation

Does the hospital have the following investigations for stroke patients:

30. Urgent CT scan on arrival at the hospital? ☐ Yes ☐ No

If YES, what is the availability?

☐ 24 hours, 7 days of the week
☐ Working hours only (8-5)
☐ Working hours (8-5) and on – call after hours

31. MR Imaging ☐ Yes ☐ No

If YES, what is the availability?

a. On-site MRI within 24-48 hours of stroke presentation ☐ Yes ☐ No

b. Off-site MRI within 24-48 hours of stroke presentation ☐ Yes ☐ No

32. Advanced imaging (CT perfusion or angiography) ☐ Yes ☐ No

If YES, what is the availability?

a. CTP on most patients ☐ Yes ☐ No

b. CTA on most patients ☐ Yes ☐ No

33. Is tele-radiology used at the hospital for rapid imaging interpretation? ☐ Yes ☐ No

34. Carotid Doppler ultrasound? ☐ Yes ☐ No

If YES, what is the availability?

a. On-site within 48 hours of presentation? ☐ Yes ☐ No

b. Off-site within 48 hours of presentation? ☐ Yes ☐ No

35. Is Transcranial Doppler routinely performed on stroke patients in the first 24 hours of admission? ☐ Yes ☐ No

36. Echocardiography (transthoracic and/or transesophageal)? ☐ Yes ☐ No

37. Holter monitor or 24hrs ECG for arrhythmia? ☐ Yes ☐ No

38. Is emergency laboratory testing available at all times? ☐ Yes ☐ No

Acute treatment for stroke

39. Do you offer intravenous thrombolysis (rt-PA) for appropriate stroke patients at the hospital? ☐ Yes ☐ No

a. If YES, when is it available?
- ☐ Available 24 hours, 7 days per week
- ☐ Monday to Friday during working hours (8-5)
- ☐ Limited service

b. Is there a standardised protocol for administering intravenous rt-PA? ☐ Yes ☐ No

c. How many patients were thrombolysed in the past 12 months? |_|_|_| patients

d. What proportions (%) of ischaemic stroke patients receive rt-PA? |_|_|_| %

e. What is the recommended time window for administering intravenous rtPA in eligible ischemic stroke patients at your hospital?
- ☐ 3 hours
- ☐ 4.5 hours
- ☐ Other, please specify:____________________

f. Do you routinely collect door-to-needle (DTN) time? ☐ Yes ☐ No

g. If YES, what is the current average DTN time for rt-PA? |_|_|_| minutes

h. Is intravenous thrombolysis performed using tele-medicine? ☐ Yes ☐ No

40. Are endovascular therapies available for stroke patients at the hospital? ☐ Yes ☐ No

a. If YES, which ones: *(tick all that apply)*
- ☐ Intra-arterial thrombolysis and/or thrombectomy of acute ischaemic stroke
- ☐ Endovascular (ie coiling) for the treatment of intracranial aneurysms
- ☐ Endovascular treatment (ie stents) for carotid stenosis

41. Is endarterectomy for carotid stenosis available at the hospital? ☐ Yes ☐ No

a. If NO, is there a transfer agreement with another hospital? ☐ Yes ☐ No

42. Is neurosurgery available onsite? ☐ Yes ☐ No

a. If NO, is there a transfer agreement with another hospital? ☐ Yes ☐ No

43. What is current practice for positioning patients in the acute (<24 hours) phase of **ischaemic stroke**? *(tick one)*
- ☐ Lying flat: 0-5 degrees *(allow head on 1 flat pillow but no elevation of head of bed)*
- ☐ Sitting up: ≥30 degrees position
- ☐ No standard positioning
- ☐ Unsure
- ☐ Other, please specify: ____________________

44. What is current practice for positioning patients in the acute (<24 hours) phase of **intracerebral haemorrhage**? *(tick one)*
- ☐ Lying flat: 0-5 degrees position *(allow head on flat pillow but no elevation of head of bed)*
- ☐ Sitting up: ≥30 degrees position
- ☐ No standard positioning
- ☐ Unsure
- ☐ Other, please specify: ____________________

45. Are there specific clinical situations in which you would alter a patient's position? ☐ Yes ☐ No

a. If yes, what is the current practice for positioning patients in the clinical situations below? *(tick all that apply)*
- ☐ Lying flat for revascularisation (iv or ia thrombolysis or clot retrieval)
- ☐ Sitting up (≥30°) for large hemispheric infarction
- ☐ Sitting up (≥30°) for mechanically ventilated patients
- ☐ Sitting up (≥30°) for drowsy or comatose patients
- ☐ Sitting up (≥30°) for patients with pneumonia
- ☐ Sitting up (≥30°) for patients with dysphagia when they are not eating or drinking (inter meals period)
- ☐ Other, please specify:

____

To answer the following questions please refer to any existing policy in any department of the hospital where a stroke patient is likely to be admitted

46. Is there a policy indicating early (<24 hours) mobilisation stroke patients (including sitting up or out of bed)? ☐ Yes ☐ No

47. Is there a policy indicating that stroke patients with a nasogastric tube should be positioned sitting up in bed? ☐ Yes ☐ No

Stroke care and Stroke Care Unit

48. Does the hospital have a special pathway, ward or service organisation for the management of patients with acute stroke? ☐ Yes ☐ No

49. Is there a dedicated stroke ward/unit? *(ie a discrete ward or section of ward caring exclusively for stroke patients)* ☐ Yes ☐ No

50. If YES, complete (a) and (b) as it best describes the type of stroke ward/unit that is in the hospital:

a. ☐ Acute stroke unit *(ie accept patients acutely but aims for early discharge (usually within seven days))*

☐ Rehabilitation stroke unit *(ie accept patients after a delay, usually 7 or more days, with a focus on rehabilitation)*

☐ Comprehensive stroke units *(ie combined acute and rehabilitation, accept patients acutely but also provides early rehabilitation for up to several weeks if necessary)*

b. ☐ '**intensive**' with continuous monitoring, high staffing levels and life support facilities

☐ **'semi-intensive'** with continuous monitoring, high staffing but no life support facilities

☐ **'non-intensive'** with none of the above.

51. Number of dedicated stroke beds in the hospital? |_|_|_|_|_| beds

52. Number of acute stroke patients admitted to the hospital annually? |_|_|_|_|_| patients

53. Number of acute stroke patients treated in the stroke care unit annually? |_|_|_|_| patients

54. Are there guidelines for acute treatment of stroke patients in the stroke care unit? ☐ Yes ☐ No

Generally, what is the number of patients to one staff? *(answer 0 if not applicable)*

55. Number of patients to one nurse |_|_|

56. Number of patients to one health assistant |_|_|

57. Is a doctor stroke specialist available in the stroke care unit? *(tick one)*

☐ Availability 24 hours, 7 days per week
☐ Monday to Friday during working hours (8-5) and on-call after hours
☐ Monday to Friday during working hours (8-5)
☐ Limited availability
☐ Not available

58. Does the stroke unit team provide medical care or advice for patients not in the stroke care ward/unit? *(i.e. for outliers, 'in-reach' or 'mobile' service)* ☐ Yes ☐ No

How many of the following medical officers attend a typical ward round in the stroke ward/unit?

		weekdays	*weekends*
59.	Consultants	|_|_|_|	|_|_|_|
60.	Fellow/advanced trainees *(senior training doctors)*	|_|_|_|	|_|_|_|
61.	Registrars *(mid-level training doctor)*	|_|_|_|	|_|_|_|
62.	Junior medical officers (residents/interns) *(junior doctors)*	|_|_|_|	|_|_|_|

63. What is the stroke specialist consultant review process of patients on the ward?

☐ 1 consultant sees all patients on a daily roster
☐ 1 consultant sees all patients on a weekly roster
☐ 1 consultant sees all patients on a 2-4 week roster
☐ Each consultant regularly sees their own patients

64. After hours, is there any special doctor rostered to be available to attend specifically to stroke patients who require admission to hospital? ☐ Yes ☐ No

a. If YES, what is their level of expertise: *(tick all that apply)*

☐ Consultant
☐ Fellow/advanced trainee
☐ Registrar
☐ Junior medical officers (residents/interns)

65. Are there other health professionals involved in the management of stroke patients at the hospital? *(tick all that apply)*

☐ Advanced medical trainee
☐ Clinical psychology
☐ Neuropsychology
☐ Dietician
☐ General physician
☐ General practitioner
☐ Geriatrician
☐ Neurologist
☐ Clinical nurse consultant (CNC)
☐ Clinical nurse specialist (CNS)
☐ Stroke care coordinator
☐ Stroke specialist research nurse
☐ Stroke nurse educator
☐ Dysphagia specialist nurse
☐ Nurse practitioner
☐ Nursing unit manager (NUM)
☐ Occupational therapist
☐ Physiotherapist
☐ Rehabilitation physician
☐ Social worker
☐ Speech language pathologist
☐ Other:______________________________

66. Is there a protocol for referral to the following health professionals? *(tick all that apply)*

- ☐ Physiotherapist
- ☐ Speech pathologist
- ☐ Occupational therapist
- ☐ Dietician
- ☐ Psychologist
- ☐ Psychiatrist
- ☐ Social worker

67. Are there multidisciplinary stroke team meetings held at least weekly? ☐ Yes ☐ No

68. Who decide the position of stroke patients in routine care? *(tick one)*

- ☐ Stroke physician or stroke registrar
- ☐ Emergency physician
- ☐ Nurses
- ☐ Health assistants
- ☐ Other, please specify: ____________________

69. Are assessment scales routinely used for any of the following situations? *(tick all that apply)*

- ☐ Consciousness level
- ☐ Motor impairment
- ☐ Visual impairment
- ☐ Sensory impairment
- ☐ Cognitive impairment
- ☐ Executive function
- ☐ Activities of daily living
- ☐ Mood
- ☐ Incontinence
- ☐ Pressure sore risk
- ☐ Nutrition

70. Are there local protocols (including assessment/monitoring) for any of the following situations? *(tick all that apply)*

- ☐ Fever control
- ☐ Reducing elevated blood glucose levels
- ☐ Swallow dysfunction

71. When is a swallowing screen performed for stroke patients? *(tick one)*

- ☐ within 24 hours for all patients
- ☐ within 24 hours for selected high risk patients
- ☐ before feeding for all patients
- ☐ before feeding for selected high risk patients
- ☐ never performed

72. Is a formal swallowing assessment by a speech pathologist or speech and language therapist performed routinely? *(tick one)*

- ☐ on every stroke patient
- ☐ on referral
- ☐ on selected high risk patients
- ☐ never performed

Discharge plans, rehabilitation and follow-up services

73. Is a discharge summary sent to the General Practitioner? ☐ Yes ☐ No

74. Do all stroke patients receive a routine outpatient clinic follow-up review? ☐ Yes ☐ No

75. Is there an Early Supported Discharge service with home-based rehabilitation and assessment? ☐ Yes ☐ No

76. Is there access to ongoing rehabilitation at the hospital or at another hospital? ☐ Yes ☐ No

77. Is there a home nursing service available for stroke patients? ☐ Yes ☐ No

78. Is there access to palliative care services? ☐ Yes ☐ No

Continuing education, research and quality improvement

79. Do nursing staff attend continuing education on stroke management? ☐ Yes ☐ No

80. In the last 2 years, has the stroke team been involved in quality improvement activities (e.g. quality assurance, local audit, developing strategies)?

- ☐ Yes
- ☐ No
- ☐ Unsure

81. How would you rate (on a scale '1-10') the support received from hospital management on quality improvement in stroke care? *(0 for none and 10 for exceptional support)* |_|_|

Thank you for you time in completing the questionnaire.
